# Multifunctional VEGF/CeO_2_-Loaded Methacrylated Chitosan Hydrogel Promotes Renal Repair Through Immune-Metabolic Reprogramming and Structural Preservation Following Ischemia–Reperfusion Injury

**DOI:** 10.3390/pharmaceutics18081025

**Published:** 2026-08-18

**Authors:** Qing Sun, Yang Fu, Tianwei Wang, Zongyuan Xu, Zeping Gui, Kun Liu, Xuzhong Liu

**Affiliations:** 1 Department of Urology, Huai’an First People’s Hospital, Nanjing Medical University, Huai’an 223300, China; sunqing215@njmu.edu.cn (Q.S.); fuyang199003@163.com (Y.F.); wtw1865@njmu.edu.cn (T.W.); xu222@163.com (Z.X.); 2 Department of Urology, The Second Affiliated Hospital of Nanjing Medical University, Nanjing 211166, China; njmugzp@gmail.com

**Keywords:** methacrylated chitosan, injectable hydrogel, cerium oxide, VEGF, renal ischemia–reperfusion injury, mixed-valence cerium oxide

## Abstract

**Background/Objectives**: Renal ischemia–reperfusion injury (IRI) and infection-associated renal damage are characterized by persistent inflammation, oxidative stress, microvascular dysfunction, and impaired tissue regeneration, creating a hostile microenvironment that limits effective repair. We developed an injectable, photocrosslinkable methacrylated chitosan (CSMA) hydrogel for the localized co-delivery of cerium oxide nanoparticles (CeO_2_NPs) and vascular endothelial growth factor (VEGF), aiming to integrate redox modulation, antibacterial activity, and regenerative support. **Methods:** Gelation, microstructure, rheology, degradation, and CeO2NP/VEGF were characterized. Tubular epithelial and fibroblast migration and endothelial network formation, angiogenic gene expression, and antibacterial activity against *Staphylococcus aureus* and *Escherichia coli* were evaluated in vitro. Theraputic performance was assessed by renal surface application in a rat renal IRI model and catheter-mediated interavsical administration in an ascending urinary tract infection model. Systematic biocompatibility was evaluated separately in a 14-day subcutaneous implantation study. Renal response were further investigated using transcriptomic and targeted molecular analyses. **Results:** The CSMA/VEGF/CeO_2_NPs hydrogel exhibited rapid in situ gelation, interconnected porous architecture, stable viscoelasticity, gradual degradation, and sustained release of both CeO_2_NPs and VEGF. The formulation enhanced tubular epithelial and fibroblast migration, promoted endothelial network formation and angiogenic gene expression and effectively inhibited both *S. aureus* and *E. coli*. In a surgically controlled rat renal IRI model, direct renal-surface application of the hydrogel reduced tubular injury, inflammatory infiltration, and fibrotic remodeling. In a separate ascending urinary tract infection model, catheter-based intravesical administration reduced the ascending renal bacterial burden and infection-associated inflammatory injury. No detectable adverse systemic effects observed under the tested conditions over the 14-day observation period in the subcutaneous implantation. Transcriptomic analyses further revealed that CSMA/VEGF/CeO_2_NPs treatment was associated with marked remodeling of the renal injury microenvironment, characterized by suppression of antigen presentation and immune activation pathways, alongside restoration of metabolic programs associated with amino acid, lipid, and purine metabolism. These molecular changes were accompanied by downregulation of CIITA/CD74/MHC-II signaling, recovery of metabolic regulators AGXT and ACOX1, modulation of Hippo/YAP- and ECM-associated pathways, and preservation of renal structural markers including nephrin and WT1. **Conclusions:** The localized CSMA-mediated co-delivery of CSMA/VEGF/CeO_2_NPs hydrogel promotes renal repair through resolution of maladaptive immune activation, metabolic reprogramming, angiogenic enhancement, and preservation of renal structural integrity, providing a promising biomaterial strategy for the treatment of ischemic and infection-associated renal injuries.

## 1. Introduction

Repairing renal tissue under injury- and contamination-prone conditions remains one of the harder problems in regenerative medicine. Damage arising from ischaemia–reperfusion, trauma, tumor-related intervention, infection, or reconstructive surgery rarely involves a single event. Instead, it triggers a series of interconnected processes, including sustained inflammatory activation, oxidative stress, microvascular dysfunction, mechanical instability, and an increased risk of bacterial colonization, all of which collectively create an environment hostile to remodeling and slow to recover [1,2]. Because the intrinsic regenerative capacity of renal tissue is limited, partly due to the absence of postnatal nephrogenesis, kidneys have a more restricted capacity for structural regeneration than highly regenerative tissues such as the liver, epidermis, and intestinal epithelium [3,4,5]. There is growing interest in biomaterials that can act on several of these processes simultaneously: filling a defect, lending temporary mechanical support, limiting bacterial burden, and steering the local environment toward repair [6,7].

From a materials standpoint, a scaffold suited to infection-prone soft-tissue repair should be injectable for minimally invasive delivery, able to gel rapidly in situ so that it conforms to irregular defects, mechanically stable enough to maintain coverage, and sufficiently hydrated and porous to allow nutrient exchange and cellular infiltration [8,9]. Because contamination and poor vascularization are the two most common barriers to healing, the ideal material would also carry both antimicrobial and pro-regenerative activity. Combining all of these attributes in one platform without compromising any of them remains difficult [10].

Chitosan-based hydrogels are attractive starting points for this purpose, owing to their biocompatibility, biodegradability, and intrinsic hemostatic and mild antibacterial activity [11,12,13,14]. Methacrylated chitosan (CSMA) is particularly useful because it can be photo-crosslinked rapidly into a stable network, which suits injectable delivery into irregular defects [15]. On its own, however, CSMA offers only modest functionality under inflammatory or contaminated conditions, which limits its usefulness in complex repair settings [16]. A productive way forward is to reinforce the network with bioactive components that each address a specific deficit [17,18,19,20,21,22].

Our design is based on a literature-informed mechanistic hypothesis supported by indirect compositional characterization and functional observations rather than direct mechanistic evidence. The CeO_2_NP component was selected because it may address two major pathological features of renal injury. In the present particles, Ce^4+^ was the predominant surface species, whereas Ce^3+^ accounted for approximately 34% of the surface cerium. Based on the previous literature, together with the present compositional characterization and antibacterial findings, the observed antibacterial activity may be associated with the predominant Ce^4+^ surface species, although this mechanism was not directly investigated in the present study. Likewise, the detected Ce^3+^ surface population and associated oxygen vacancies may contribute to ROS-modulating activity, as suggested by previous studies. The design additionally incorporates the vascular endothelial growth factor (VEGF), the principal driver of endothelial activation and neovascularization, the efficacy of which depends heavily on localized retention and sustained delivery.

Most reported hydrogels address antimicrobial activity and growth-factor delivery separately and seldom combine these functions within a single platform [23]. We therefore developed an injectable CSMA hydrogel that co-delivers CeO_2_NPs and VEGF, unifying putative ROS-modulating activity, antibacterial activity, and pro-angiogenic signaling through a single design. The present work is intended as a proof of concept rather than a definitive reconstruction study: using a renal ischemia–reperfusion model and a complementary kidney infection model, we ask whether this multifunctional hydrogel can protect renal tissue, lower bacterial burden, and remodel the injury microenvironment toward repair.

## 2. Materials and Methods

### 2.1. Materials

Low-molecular-weight chitosan (Mw ≈ 100 kDa, 85–90% deacetylated) and lithium phenyl (2, 4, 6-trimethylbenzoyl) phosphinate (LAP) were purchased from Aladdin Reagent Co., Ltd. (Shanghai, China). Cerium nitrate hexahydrate (Ce(NO_3_)_3_·6H_2_O, ≥99.0%) and sodium hydroxide (NaOH, ≥98%) were obtained from Sigma-Aldrich (St. Louis, MO, USA). Recombinant human vascular endothelial growth factor-165 (VEGF-165) was purchased from Novoprotein (Suzhou, China). Other analytical-grade reagents were supplied by Macklin Biochemical Co., Ltd. (Shanghai, China) and used as received.

Dulbecco’s modified Eagle’s medium (DMEM), keratinocyte serum-free medium (K-SFM), fetal bovine serum (FBS), penicillin-streptomycin solution (10,000 U mL^−1^), and 0.25% trypsin-EDTA were purchased from Gibco (Thermo Fisher Scientific, Waltham, MA, USA). The immortalized human proximal tubular epithelial cell line HK-2, mouse fibroblast cell line NIH 3T3, and human umbilical vein endothelial cells (HUVECs) were obtained from the Cell Bank of the Chinese Academy of Sciences (Shanghai, China). All solutions were prepared using ultrapure water.

### 2.2. Synthesis of Methacrylated Chitosan (CSMA)

Methacrylated chitosan (CSMA) was synthesized by reacting chitosan with methacrylic anhydride. Briefly, chitosan (5 g) was dissolved in 500 mL of 0.1 M acetic acid, and the pH was adjusted to 8.0–8.5 using 2 M NaOH under ice-bath conditions. The mixture appeared as a fine, partially swollen suspension. Methacrylic anhydride (5 mL) was added dropwise over 30 min under a nitrogen atmosphere, and the reaction was allowed to proceed for 24 h at 4 °C with continuous stirring. The obtained solution was dialyzed against deionized water using dialysis tubing with a molecular weight cut-off of 12–14 kDa for 3 days, followed by freeze-drying. The resulting CSMA was stored at −20 °C until use. The degree of methacrylation was approximately 22%, as determined by ^1^H NMR spectroscopy.

### 2.3. Preparation of Nanosized Cerium Oxide Nanoparticles (CeO_2_NPs)

Nanosized cerium oxide nanoparticles (CeO_2_NPs) were prepared using a chemical precipitation method. Briefly, cerium nitrate hexahydrate was dissolved in deionized water to obtain 100 mL of a 1 mM precursor solution under continuous magnetic stirring at room temperature. NaOH solution was then added dropwise until the pH of the reaction system reached 9–10, resulting in the formation of a light-yellow suspension. The reaction mixture was stirred for 2 h and subsequently aged for 12 h to allow complete nucleation and growth of the nanoparticles.

The obtained nanoparticles were collected by centrifugation, washed three times with deionized water and ethanol to remove unreacted precursors and residual ions, and then redispersed in deionized water. Polyvinylpyrrolidone (PVP, 10 mg mL^−1^) was added as a stabilizing agent to improve colloidal dispersion. Transmission electron microscopy (TEM) revealed individual primary particles with diameters of 20–30 nm; the larger value of 123.7 ± 8.1 nm obtained from size distribution measurements reflects partial aggregation of primary particles in aqueous suspension, which is commonly observed for nanosized oxide nanoparticles even under stabilized conditions. PVP was added as a stabilizing agent to limit further aggregation, and the colloid was stored at 4 °C in the dark and used within one week to minimize aggregation-induced changes in particle properties. The morphology and size of the CeO_2_NPs were examined using transmission electron microscopy (TEM; JEM-2100, JEOL Ltd., Tokyo, Japan, operated at 200 kV). The particle size distribution was obtained by measuring at least 100 individual nanoparticles from TEM images using ImageJ software (version 1.53t, National Institutes of Health, Bethesda, MD, USA). The crystalline phase was identified using X-ray diffraction (XRD; D8 Advance, Bruker AXS GmbH, Karlsruhe, Germany, Cu Kα radiation, λ = 1.5406 Å) across a 2θ range of 10–90°. The surface elemental composition and Ce^3+^/Ce^4+^ oxidation states were analyzed using X-ray photoelectron spectroscopy (XPS; AXIS Ultra, Kratos Analytical Ltd., Manchester, UK) with a monochromatic Al Kα source). The binding energies were calibrated to the adventitious C 1s peak at 284.8 eV and the Ce 3d region was used to assess the relative Ce^3+^ fraction.

### 2.4. Fabrication of the CSMA/VEGF/CeO_2_NPs Hydrogel

CSMA was dispersed in PBS at 3% (*w*/*v*) to form a visually homogeneous colloidal precursor rather than a fully dissolved solution. LAP (0.15% *w*/*v*) was then added as the photoinitiator. CeO_2_NPs colloid was added to achieve a final concentration of 60 μg mL^−1^, and recombinant VEGF-165 was gently incorporated at a final concentration of 1000 ng mL^−1^ under ice-bath conditions. The precursor appeared visually homogeneous and was injected into molds or applied directly onto tissue defects and then crosslinked by irradiation with 405 nm violet light for 30 s to form a stable hydrogel. CSMA, CSMA/VEGF, CSMA/CeO_2_NPs, and CSMA/VEGF/CeO_2_NPs hydrogels were prepared using the same procedure and used for subsequent experiments.

### 2.5. Physical Characterization of the CSMA/VEGF/CeO_2_NPs Hydrogel

#### 2.5.1. Morphology

The internal morphology of the hydrogels was characterized by scanning electron microscopy (SEM; SU8010, Hitachi High-Technologies Corporation, Tokyo, Japan). Hydrogel samples were frozen, lyophilized, and fractured to expose cross-sections. The samples were then sputter-coated with gold and observed using field-emission SEM at an accelerating voltage of 5 kV.

#### 2.5.2. Equilibrium Swelling Ratio

Cylindrical hydrogel samples with a diameter of 8 mm and a height of 2 mm were lyophilized and weighed to obtain the initial dry mass (m_0_). The samples were then immersed in PBS (pH 7.4) at 37 °C. At predetermined time points, the samples were removed, gently blotted to remove surface moisture, and weighed to obtain the swollen mass (m_t_). The swelling ratio (SR) was calculated according to the following equation: SR (%) = (mt − m_0_)/m_0_ × 100%.

#### 2.5.3. Mechanical and Rheological Properties

Unconfined compression tests were performed using a universal testing machine (5944, Instron, Norwood, MA, USA) equipped with a 1 kN load cell. Cylindrical hydrogel samples were compressed at a rate of 1 mm min^−1^ to 40% strain. The compressive Young’s modulus was calculated from the linear region of the stress–strain curve between 5% and 15% strain.

The viscoelastic properties of the hydrogels were measured using a rotational rheometer with a 20 mm parallel-plate geometry at 25 °C and a 1 mm gap. Time-sweep tests were performed at 1% strain and 1 rad s^−1^. Frequency-sweep measurements were conducted from 0.1 to 100 rad s^−1^ at 1% strain. Strain-recovery behavior was assessed using a step-strain protocol alternating between 1% and 300% strain.

#### 2.5.4. In Vitro Degradation

Hydrogel samples were lyophilized and weighed to obtain the initial dry weight (W_0_). The samples were then incubated in 10 mL of PBS (pH 7.4) at 37 °C under gentle shaking for 6 days. At predetermined intervals, the samples were removed, rinsed with deionized water, lyophilized again, and weighed to determine the remaining dry weight (W_t_). The mass remaining was calculated according to the following equation: Mass remaining (%) = W_t_/W_0_ × 100%.

### 2.6. Cerium Release and VEGF Release Evaluation

#### 2.6.1. Cerium Release

To evaluate the release behavior of cerium from the hydrogels, CeO_2_NPs-containing hydrogels were prepared as described above and immersed in 2 mL of PBS at 37 °C under gentle shaking (*n* = 3). At predetermined time points on days 1, 3, 5, 7, 10, and 14, 1 mL of release medium was collected and replaced with an equal volume of fresh PBS. The amount of released cerium in the collected samples was quantified by inductively coupled plasma optical emission spectroscopy (ICP-OES; 5110, Agilent Technologies, Santa Clara, CA, USA). Cumulative cerium release was calculated based on the initial cerium loading in the hydrogel.

#### 2.6.2. VEGF Release

VEGF-containing hydrogels were incubated in 2 mL of PBS supplemented with 0.1% bovine serum albumin (BSA) at 37 °C under gentle shaking (*n* = 3). At predetermined time points on days 1, 3, 5, 7, 10, and 14, 1 mL of release medium was collected and replaced with an equal volume of fresh medium. VEGF concentration in the collected samples was measured using a human VEGF ELISA kit according to the manufacturer’s instructions. Cumulative VEGF release was expressed as the percentage of the initially loaded VEGF.

### 2.7. In Vitro Cellular Experiments

All cell experiments were performed in triplicate unless otherwise stated. Hydrogel extracts were prepared by incubating hydrogels in complete culture medium at an extraction ratio of 1 g hydrogel to 10 mL medium.

#### 2.7.1. Cell Migration

Tubular epithelial restitution was evaluated using a scratch-wound assay in HK-2 cells. Fibroblast migration and matrix-remodeling capacity were assessed identically in NIH 3T3 cells. Briefly, cells were seeded in culture plates and grown to confluent monolayers. A linear scratch was created using a sterile pipette tip, and detached cells were removed by washing with PBS. The cells were then treated with hydrogel extracts and imaged at 0, 6, and 24 h. Gap closure was quantified using ImageJ.

#### 2.7.2. Angiogenesis

The pro-angiogenic activity of the hydrogels was evaluated using a HUVEC tube formation assay. HUVECs were seeded onto growth-factor-reduced Matrigel and treated with different hydrogel extracts. After incubation for 4–6 h, cells were stained with calcein-AM (Beyotime Biotechnology, Shanghai, China) and imaged using a fluorescence microscope (IX73, Olympus, Tokyo, Japan). Total tube length and branch points were quantified using AngioTool (version 0.6a, National Cancer Institute, Bethesda, MD, USA).

The expression of angiogenesis-related genes, including Pecam1, Vegfa, and Il1b, was measured by quantitative real-time PCR (qPCR Applied Biosystems, Waltham, MA, USA), with GAPDH used as the reference gene. Relative gene expression was calculated using the 2^−ΔΔCt^ method. Pro-angiogenic activity was further evaluated using a chick chorioallantoic membrane (CAM) assay.

#### 2.7.3. Biocompatibility

Cytocompatibility was assessed in HUVECs and NIH 3T3 cells using live/dead staining and the Cell Counting Kit-8 (CCK-8; Beyotime Biotechnology, Shanghai, China) assay on days 1, 3, and 7. For live/dead staining, cells were treated with hydrogel extracts and then stained according to the manufacturer’s instructions.

Hemocompatibility was evaluated using a hemolysis assay with rat erythrocytes. Briefly, erythrocyte suspensions incubated with hydrogel samples or hydrogel extracts at 37 °C for 2 h. Distilled water and PBS were used as positive and negative controls, respectively. The absorbance of the supernatant was measured at 540 nm, and the hemolysis ratio was calculated accordingly.

### 2.8. Antibacterial Activity

The antibacterial activity of the hydrogels was evaluated against *Staphylococcus aureus* and *Escherichia coli*. Agar disk diffusion assays were performed using hydrogel disks with a diameter of 8 mm, and the inhibition zones were measured using a caliper. Bacterial growth kinetics were assessed by monitoring the optical density at 600 nm (OD_600_) over time. In addition, colony-forming-unit (CFU) quantification was performed after 24 h of incubation. Antibacterial efficiency was expressed relative to the CSMA group.

### 2.9. In Vivo Animal Experiments

All animal procedures were approved by the Animal Research Ethics Committee of Nanjing Medical University (IACUC-2109025) and conducted in accordance with the ARRIVE guidelines. Male Sprague-Dawley (SD) rats were used for all in vivo experiments. Animals were housed under standard laboratory conditions with free access to food and water. Anesthesia, postoperative analgesia, and euthanasia were performed according to institutional guidelines. Rats were anesthetized by intraperitoneal injection of sodium pentobarbital (40 mg kg^−1^); at the experimental endpoint, animals were euthanized by an overdose of sodium pentobarbital followed by cervical dislocation.

#### 2.9.1. Fourteen-Day Systemic Evaluation

To assess systemic biocompatibility, hydrogel disks were implanted subcutaneously into male SD rats (*n* = 6). After 14 days, major organs, including the heart, liver, spleen, lung, and kidney, were harvested for hematoxylin and eosin (H&E) staining. Body weight was recorded at regular intervals, and arterial oxygen saturation (SpO_2_) was measured using a small-animal pulse oximeter (MouseOx Plus, STARR Life Sciences Corp., Oakmont, PA, USA). Heart rate and body temperature were monitored at matched time points throughout the study to evaluate systemic physiological responses (*n* = 6).

#### 2.9.2. Hematology and Blood Biochemistry

To complement organ histology, whole blood and serum were collected for a complete blood count, including white blood cell count (WBC), hemoglobin (HGB), and platelet count (PLT), and for a serum biochemical panel including alanine aminotransferase (ALT) as a hepatic marker and blood urea nitrogen (BUN) together with serum creatinine (Scr) as renal-function markers.

#### 2.9.3. Renal Ischemia–Reperfusion Injury (IRI)

A unilateral renal ischemia–reperfusion injury model with direct renal-surface hydrogel administration was established in SD rats. Briefly, the renal pedicle was exposed and clamped for 30 min, followed by reperfusion. Sham-operated animals underwent identical surgical exposure without vascular clamping. Animals were randomly assigned to the following six groups: sham, IRI, IRI + CSMA, IRI + CSMA/VEGF, IRI + CSMA/CeO_2_NPs, and IRI + CSMA/VEGF/CeO_2_NPs (*n* = 5 per group).

Immediately after reperfusion, 0.2 mL of hydrogel precursor solution was applied to the renal surface and crosslinked in situ. At days 3, 7, and 14 after surgery, kidneys were harvested for histological and molecular analyses. H&E staining was performed to assess tubular injury, and Masson’s trichrome staining was used to evaluate collagen deposition. Tubular injury and inflammatory infiltration were semi-quantitatively scored on a 0–4 scale by blinded investigators. The collagen-positive area was quantified using ImageJ. Inflammatory/injury-related genes and vascular/repair-related genes were measured by qPCR.

#### 2.9.4. Kidney-Infection Model

A separate ascending UTI model using catheter-based intravesical hydrogel administration was established to test whether intravesical hydrogel treatment could limit infection-associated renal injury. Uropathogenic *E. coli* was introduced by transurethral inoculation to establish a bladder-centered infection that ascends to the kidney. Briefly, rats were anesthetized, and a bacterial suspension containing 1 × 10^8^ CFU mL^−1^ uropathogenic *E. coli* was instilled transurethrally at a volume of 100 μL per animal. Thirty minutes after inoculation, 0.2 mL of hydrogel precursor was instilled into the bladder, the primary site of bacterial colonization, and crosslinked in situ by transcutaneous 405 nm irradiation applied over the lower abdomen for 30 s; control animals received an equal volume of sterile PBS without hydrogel. By forming a local depot at the bladder, the hydrogel was intended to reduce the ascending bacterial reservoir and thereby limit secondary bacterial spread and injury to the kidney.

To ensure comparability with the IRI model, the same blinded scoring rubric was applied, with tubular injury and inflammatory infiltration scored from 0 to 4. At 14 days after intravesical treatment, the animals were euthanized, and the bladder, representing the primary infection site, and the kidney, representing the ascending target, were harvested for histological and RT-qPCR analyses. Renal bacterial burden was quantified by CFU counting to assess ascending renal involvement, and a matched RT-qPCR panel was used to evaluate inflammation- and repair-related gene expression in both tissues.

### 2.10. Transcriptomics and Validation

Bulk RNA sequencing was performed on renal tissues from both the infection and renal IRI models. In each model, untreated injury was compared with CSMA/VEGF/CeO_2_NPs treatment. Because only the complete formulation was sequenced, the transcriptomic changes cannot be attributed separately to CeO_2_NPs or VEGF. This is consistent with the intended design, in which CeO_2_NPs and VEGF act complementarily. CeO_2_NPs may participate in redox-associated and immune-metabolic modulation, whereas VEGF may enhance angiogenic repair responses.

Total RNA was extracted using TRIzol reagent, and RNA integrity was verified before library preparation. Poly(A) + RNA libraries were constructed and subjected to Illumina paired-end sequencing (Illumina, Inc., San Diego, CA, USA).

Raw sequencing reads were aligned to the reference genome using HISAT2 (version 2.2.1). Gene expression levels were quantified using featureCounts (version 2.0.3), and differential expression analysis was performed using DESeq2 (version 1.40.2). Genes with an adjusted *p* value < 0.05 were considered differentially expressed. Gene Ontology (GO) and Kyoto Encyclopedia of Genes and Genomes (KEGG) enrichment analyses were performed using standard bioinformatics pipelines.

To validate the sequencing results at the gene and protein levels, selected differentially expressed genes were confirmed by RT-qPCR. Key pathway-related proteins were examined by Western blotting, including the antigen-presentation regulators CIITA, MHC class II, and CD74; the Hippo/YAP- and extracellular matrix-associated proteins YAP, phospho-YAP, CTGF, and fibronectin; and the renal structural markers nephrin, WT1, and KIM-1.

### 2.11. RT-qPCR and Western Blot Validation

To validate the transcriptomic findings at both the gene and protein levels, qPCR and Western blot analyses were performed on independent renal tissue samples from the renal IRI model (*n* ≥ 3 per group). For qPCR, total RNA was reverse-transcribed and amplified using rat-specific primers, with Gapdh as the reference gene; relative expression was calculated by the 2^−ΔΔCt^ method; the validated qPCR targets were the inflammatory genes Il1b and Tnf and the metabolic genes Agxt and Acox1. For Western blot, total protein was separated by SDS-PAGE, transferred to PVDF membranes, and probed with primary antibodies against MHC class II (I-A/I-E), CD74, CIITA, YAP, phospho-YAP (Ser127), CTGF, fibronectin, nephrin, WT1, and KIM-1, with GAPDH or β-actin as loading controls. Bands were visualized by enhanced chemiluminescence and quantified by densitometry using ImageJ.

### 2.12. Statistical Analysis

Data are presented as mean ± standard deviation (SD). Statistical analyses were performed using OriginPro 2024 (OriginLab Corporation, Northampton, MA, USA). For comparisons among multiple groups, one-way analysis of variance (ANOVA) followed by Tukey’s post hoc test was used. For experiments involving multiple treatment groups and time points, two-way ANOVA followed by Tukey’s post hoc test was applied where appropriate. A value of *p* < 0.05 was considered statistically significant.

## 3. Results and Discussion

### 3.1. Synthesis and Characterization of Nanosized CeO_2_ Nanoparticles

**Figure 1:**
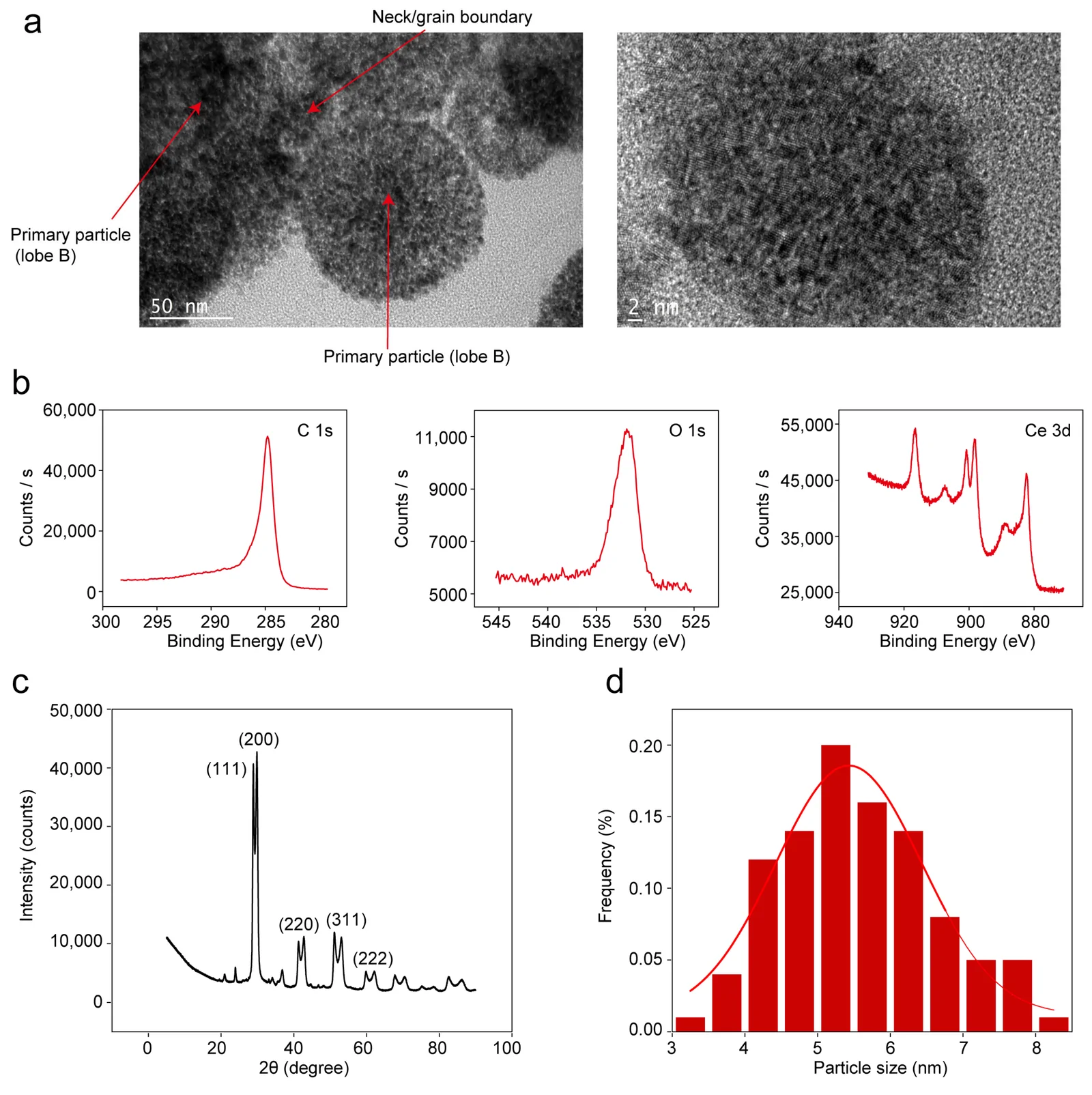
Synthesis and characterization of nanosized CeO_2_ nanoparticles. (**a**) Representative TEM images of CeO_2_NPs at low (scale bar, 50 nm) and high (scale bar, 2 nm) magnification. (**b**) XPS spectra of the CeO_2_NPs, including the C 1s, O 1s, and Ce 3d core-level regions; the Ce 3d multiplet indicates a mixed-valence Ce^3+^/Ce^4+^ surface, with a Ce^3+^ fraction of approximately 34%. This compositional finding provides indirect support for the literature-informed hypothesis of oxygen-vacancy-associated redox activity but does not directly demonstrate oxygen vacancies or redox function. (**c**) XRD pattern of the CeO_2_NPs, with reflections indexed to the cubic fluorite structure of CeO_2_. (**d**) Particle-size distribution histogram reflecting aggregate size in aqueous suspension (mean 123 ± 8.1 nm; *n* = 100); individual primary particles measured 20–30 nm by TEM, consistent with the nanosized designation used throughout this work.

Transmission electron microscopy revealed quasi-spherical cerium oxide (CeO_2_) nanoparticles with primary particle diameters of 20–30 nm, though partial aggregation was observed in aqueous suspension, consistent with the tendency of nanosized oxide nanoparticles to form clusters under aqueous conditions (Figure 1a), with individual primary particles of 20–30 nm as determined from TEM images; the measured size distribution of 123 ± 8.1 nm (Figure 1d) reflects aggregated clusters rather than discrete primary particles, consistent with the tendency of nanosized oxide nanoparticles to form aggregates in aqueous suspension. This small size provides a high surface-to-volume ratio and abundant surface sites, favoring interfacial interactions. X-ray photoelectron spectroscopy confirmed the surface composition and oxidation states of the nanoparticles (Figure 1b). The core-level scans showed the expected C 1s, O 1s, and Ce 3d signals. Peak fitting of the Ce 3d multiplet confirmed a mixed-valence surface with a Ce^3+^ fraction of approximately 34% and Ce^4+^ as the dominant species, consistent with the thermodynamically stable fluorite phase identified by XRD. X-ray diffraction further confirmed the crystalline phase, with reflections indexed to the (111), (200), (220), (311), and (222) planes of the cubic fluorite structure of CeO_2_ (Figure 1c). The broadened reflections are consistent with the nanosized crystallite size observed by TEM. Together, these results confirm the successful preparation of nanosized, mixed-valence, fluorite-phase CeO_2_NPs suitable for integration into the multifunctional hydrogel.

### 3.2. Synthesis and Structural Characterization of CSMA/VEGF/CeO_2_NPs Hydrogels

**Figure 2:**
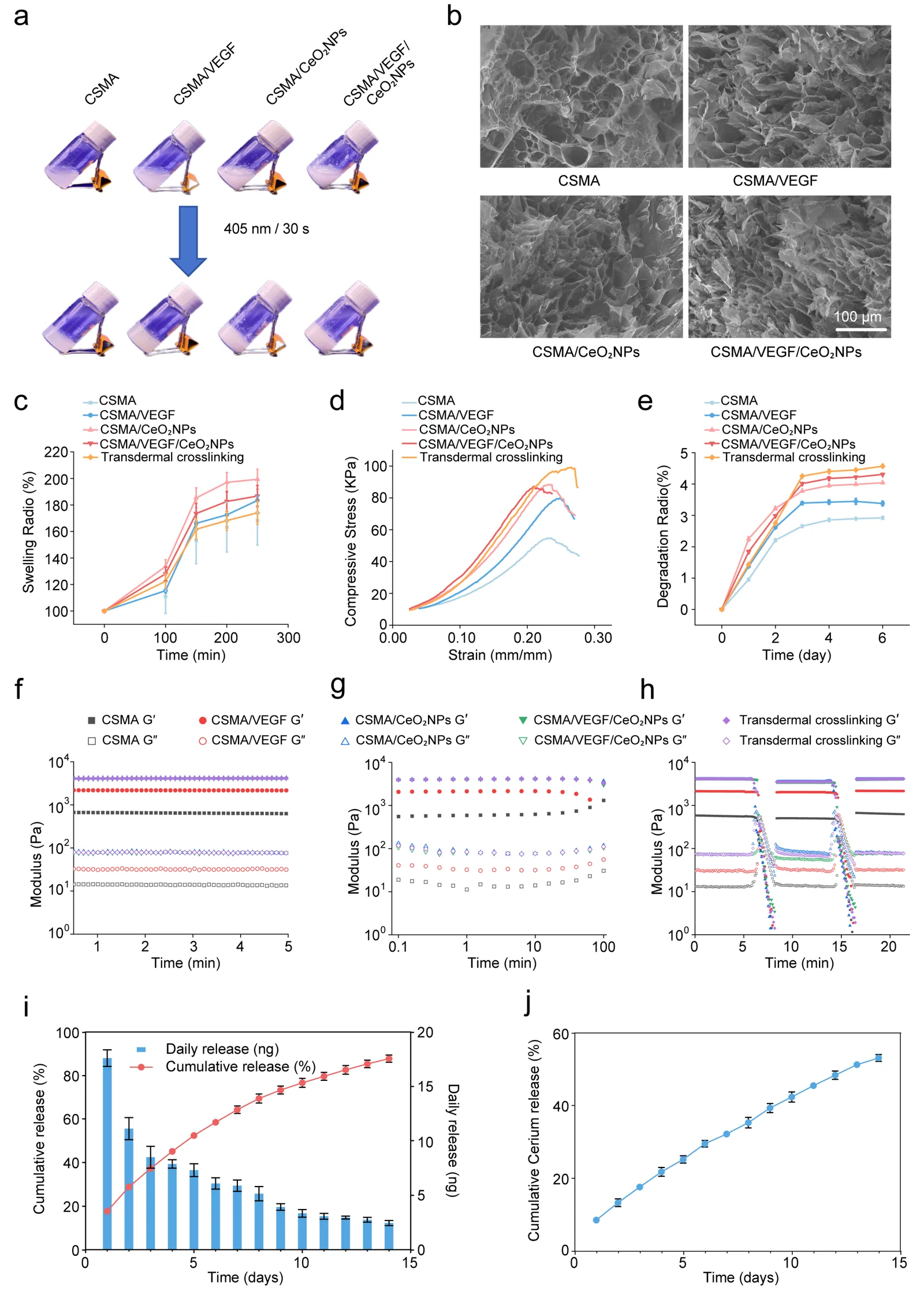
Fabrication and physicochemical characterization of CSMA-based multifunctional hydrogels. (**a**) Schematic illustration of 405 nm violet-light-induced in situ gelation of CSMA-based hydrogels. (**b**) SEM micrographs showing the interconnected porous microarchitecture of the hydrogels. (**c**) Swelling behavior of hydrogels in PBS at 37 °C. (**d**) Compressive stress–strain curves of the hydrogels. (**e**) In vitro degradation profiles of hydrogels in PBS. (**f**) Time-sweep rheological analysis showing storage modulus (G′) and loss modulus (G″) as a function of time. (**g**) Frequency-sweep rheological analysis showing G′ and G″ as a function of angular frequency. (**h**) Step-strain measurements demonstrating rapid recovery of viscoelastic properties under alternating low (1%) and high (300%) strain. (**i**) VEGF release profile from VEGF-loaded hydrogels, presented as cumulative release and daily release using dual y-axes. (**j**) Cumulative cerium release profile from CeO_2_NPs-containing hydrogels over the observation period. Data are presented as mean ± SD (*n* = 3).

All four hydrogel formulations began to gel within seconds of 405 nm violet-light irradiation and cured into coherent networks (Figure 2a), indicating that the incorporation of CeO_2_NPs and VEGF did not impair photocrosslinking of the CSMA backbone. Rapid in situ gelation was observed in all groups, suggesting that the precursors can be retained locally and conform to irregular tissue surfaces before crosslinking.

SEM observations showed that all hydrogels possessed an open, interconnected porous microstructure, with pore sizes of approximately 50–120 μm (Figure 2b). Such an architecture is beneficial for fluid transport, nutrient diffusion, cellular infiltration, and matrix remodeling during soft-tissue repair [24]. Importantly, incorporation of the inorganic filler and bioactive cargos did not disrupt pore interconnectivity, indicating good compatibility between the polymer matrix and the functional additives.

Swelling analysis indicated that the hydrogels maintained adequate water uptake in PBS at 37 °C (Figure 2c), helping to preserve a hydrated microenvironment conducive to cell survival and tissue coverage. Compression testing showed that all hydrogels retained overall elasticity, with compressive stress increasing approximately linearly up to about 23% strain (Figure 2d). Minor differences in compressive stress among formulations were observed, likely reflecting the additional physical interactions introduced by CeO_2_NPs and VEGF within the polymer network; nonetheless, all formulations maintained favorable mechanical stability and sufficient compliance for tissue integration. In the degradation assay, all hydrogels reached a quasi-equilibrium mass by day 3 and showed only limited further mass loss (<5%) over the observation period (Figure 2e), indicating adequate structural stability for initial tissue support. It should be noted that the mass-loss assay reflects primarily the elution of soluble low-molecular-weight fragments and uncrosslinked components rather than bulk polymer-chain scission; accordingly, the slow mass loss is not inconsistent with the sustained release of CeO_2_NPs and VEGF observed over 14 days (Figure 2i,j), which proceeds predominantly via diffusion through the intact porous network rather than degradation-driven matrix erosion [25]. The degradation profile was characterized over 6 days to capture the critical early structural stabilization period, whereas release profiles were extended to 14 days to fully characterize the sustained delivery kinetics; these two assays address distinct material properties and their different observation windows reflect their respective experimental purposes.

Rheological analysis further confirmed robust network formation. Time-sweep measurements showed that the storage modulus (G′) consistently exceeded the loss modulus (G″) by approximately one order of magnitude and remained stable over 300 s (Figure 2f), demonstrating dominant elastic behavior and rapid network stabilization. Frequency sweeps showed nearly frequency-independent G′ plateaus, supporting a stable crosslinked network (Figure 2g). In step-strain tests, all formulations rapidly recovered G′ after repeated exposure to high strain (300%), with more than 90% modulus recovery within 30 s (Figure 2h). This reversible viscoelastic response indicates that the hydrogels can tolerate transient deformation during handling and application and rapidly recover their structural integrity.

Overall, these physicochemical data indicate that multifunctional modification endowed the CSMA hydrogel with additional biological functionality while preserving its basic structural integrity and fundamental properties. These characteristics establish an essential materials basis for the antibacterial and pro-regenerative performance evaluated below.

### 3.3. VEGF and CeO_2_NPs Release Behavior

To help interpret the antibacterial and pro-angiogenic functions of the multifunctional hydrogel, the release behaviors of CeO_2_NPs and VEGF were evaluated over 14 days.

VEGF-loaded hydrogels showed time-dependent VEGF release (Figure 2i), characterized by an initial faster release phase followed by slower, sustained release over the subsequent days. This profile is favorable for localized angiogenic stimulation, because it can support early endothelial activation while maintaining continued VEGF availability during later stages of tissue remodeling; the bioactivity of the released VEGF is supported by the tube-formation and CAM results presented below.

The CeO_2_NP-containing formulations exhibited a gradual, sustained release profile without a pronounced initial burst (Figure 2j). This profile may support sustained local cerium availability; however, neither release–activity nor release–toxicity relationships were directly evaluated in the present study.

Taken together, these results indicate that the hydrogel serves as a reservoir for sustained local delivery of CeO_2_NPs and VEGF, with release behavior consistent with the antibacterial and pro-regenerative effects evaluated in subsequent experiments.

### 3.4. Cell Migration and Pro-Angiogenic Responses Relevant to Renal Tissue Recovery

**Figure 3:**
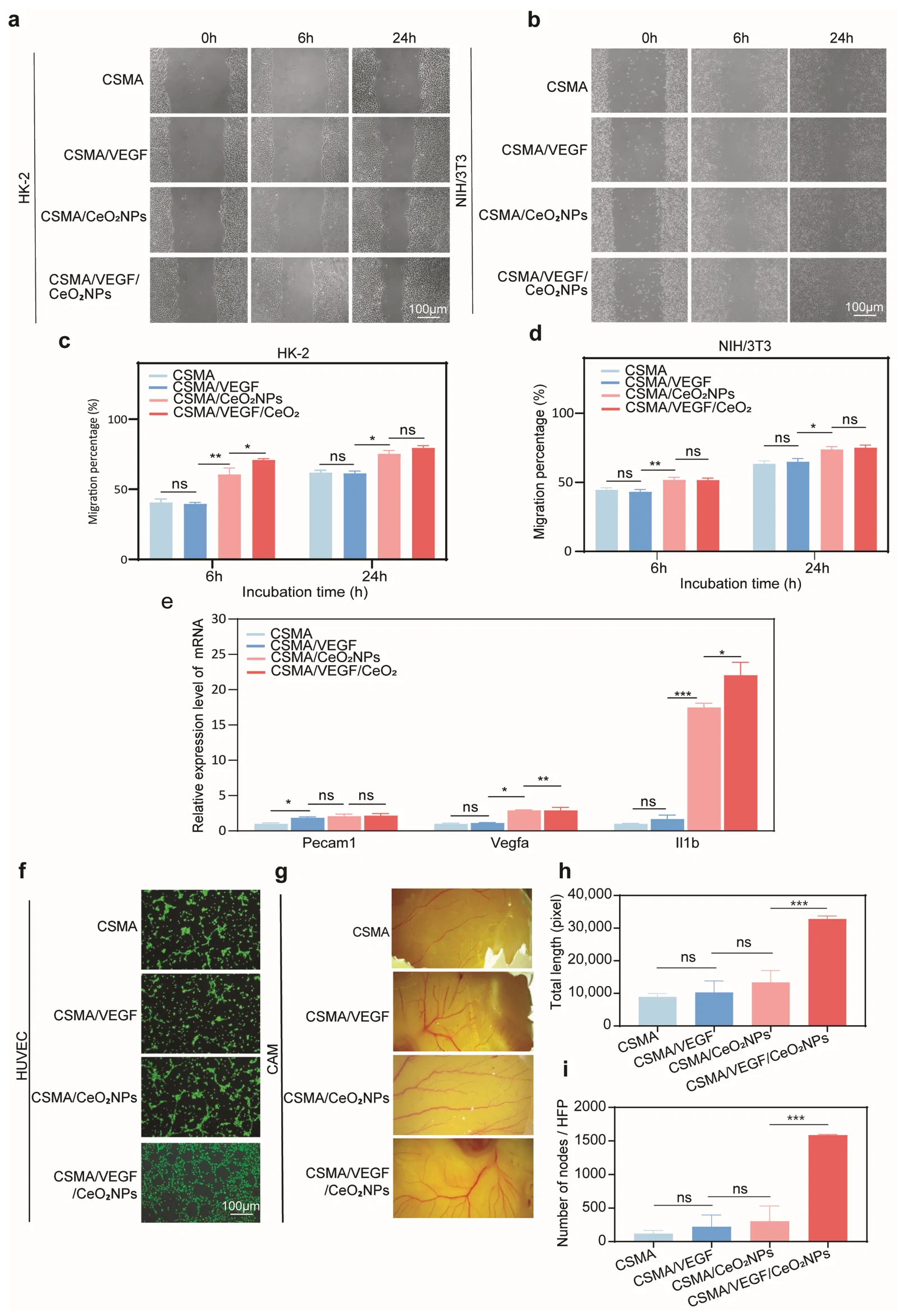
Cell migration and pro-angiogenic responses of hydrogel-conditioned media. (**a**) Representative scratch-wound images of HK-2 tubular epithelial cells at 0, 6, and 24 h after treatment with conditioned media from each hydrogel formulation. Scale bar, 200 μm. (**b**) Representative scratch-wound images of NIH 3T3 fibroblasts at 0, 6, and 24 h under the same treatment conditions. Scale bar, 200 μm. (**c**) Quantitative gap closure ratio in HK-2 cells; closure increased from ~61% (CSMA) to ~80% (CSMA/VEGF/CeO_2_NPs) at 24 h. (**d**) Quantitative gap closure ratio in NIH 3T3 cells, with near-complete closure in the CSMA/VEGF/CeO_2_NPs group at 24 h. (**e**) Relative mRNA expression of endothelial markers Pecam1, Vegfa, and Il1b in HUVECs, showing the strongest upregulation in the CSMA/VEGF/CeO_2_NPs group. (**f**) Representative fluorescence images of HUVEC tube formation on Matrigel after 4–6 h; calcein-AM staining (green). Scale bar, 200 μm. (**g**) Representative CAM assay photographs showing enhanced neovascularization in the CSMA/VEGF/CeO_2_NPs group. (**h**) Total tube length quantified by AngioTool, significantly increased in VEGF-containing groups. (**i**) Branch-point number quantified by AngioTool, confirming superior pro-angiogenic activity of the CSMA/VEGF/CeO_2_NPs formulation. Data are presented as mean ± SD (*n* = 3); * *p* < 0.05, ** *p* < 0.01, *** *p* < 0.001.

Renal recovery depends on coordinated epithelial restitution, fibroblast-driven matrix deposition, and microvascular restoration [26]. In scratch-wound assays, tubular epithelial gaps in HK-2 monolayers closed significantly faster when treated with CSMA/CeO_2_NPs and CSMA/VEGF/CeO_2_NPs extracts than with pristine CSMA (Figure 3a,c). At 24 h, gap closure increased from approximately 61% in the CSMA group to 74% in the CSMA/CeO_2_NPs group and 80% in the CSMA/VEGF/CeO_2_NPs group. Fibroblast migration, assessed in NIH 3T3 cells, showed a similar trend, with near-complete closure in the CSMA/VEGF/CeO_2_NPs group at 24 h compared with a persistent open margin in the CSMA group (Figure 3b,d).

Pro-angiogenic responses were also markedly enhanced by the VEGF-containing hydrogel extracts. In HUVECs, expression of the endothelial and angiogenesis-related markers Pecam1, Vegfa, and Il1b was upregulated relative to the CSMA group, with the strongest induction in the CSMA/VEGF/CeO_2_NPs group (Figure 3e). Tube-formation assays revealed denser, more interconnected capillary-like networks for the CSMA/VEGF/CeO_2_NPs composite, reflected by increases in both total tube length and branch-point number (Figure 3f,h,i). These in vitro findings were corroborated by the chick chorioallantoic membrane (CAM) assay, which showed enhanced neovascularization in the CSMA/VEGF/CeO_2_NPs group (Figure 3g). Notably, the migration benefit was already evident with CSMA/CeO_2_NPs, whereas the angiogenic responses depended primarily on VEGF, consistent with the complementary roles of the two components in the combined formulation. Together, these results indicate that the multifunctional hydrogel supports key cellular processes relevant to renal tissue repair, including tubular epithelial restitution, fibroblast migration, and microvascular regeneration, highlighting its potential to promote renal recovery in vivo.

### 3.5. In Vitro Compatibility and In Vivo 14-Day Systemic Evaluation

Biocompatibility was evaluated using in vitro cytocompatibility and hemocompatibility assays, together with a separate 14-day subcutaneous implantation study designed to assess potential short-term systemic effects [27].

**Figure 4:**
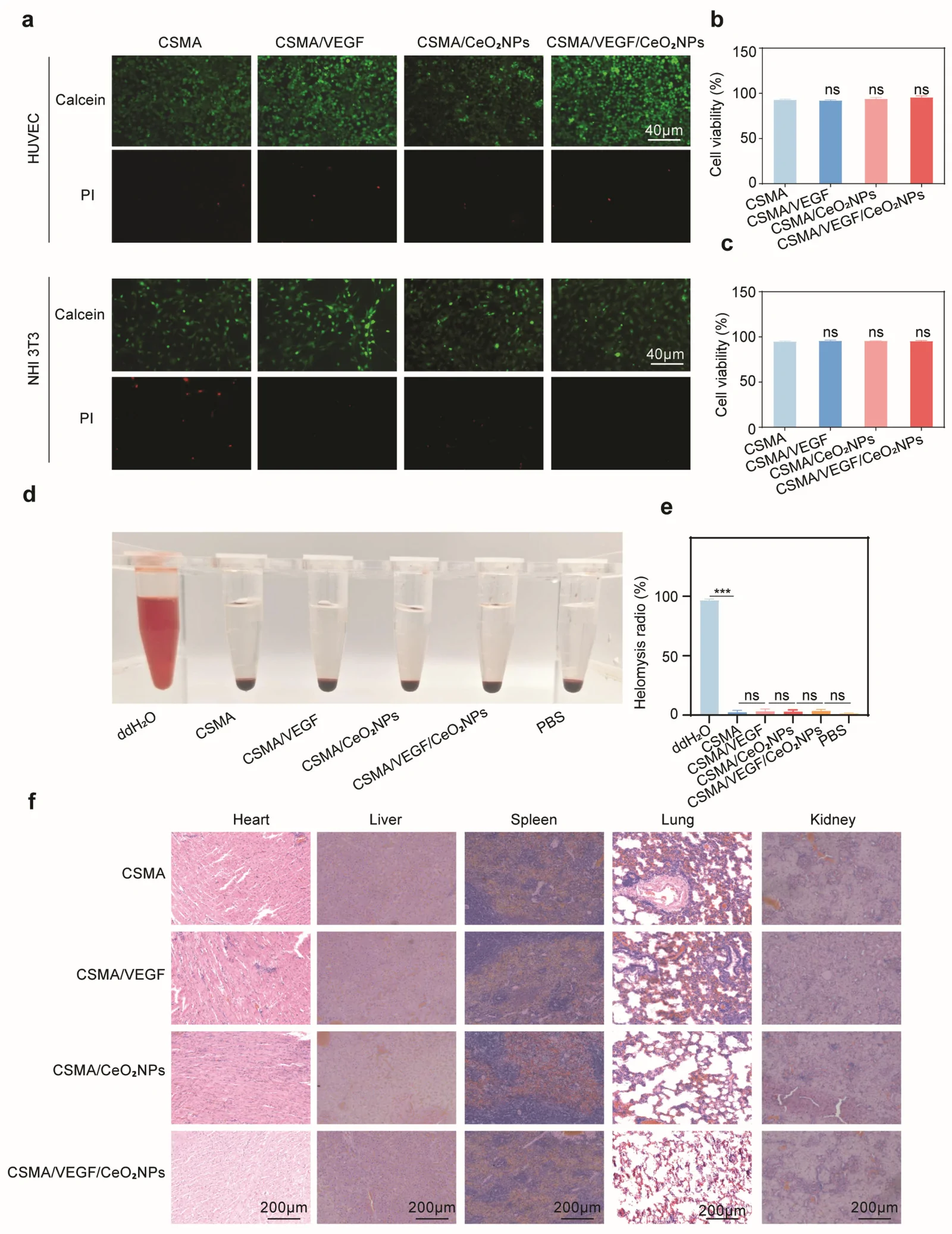
In vitro compatibility assays and organ histology following 14-day subcutaneous implantation of CSMA-based hydrogels. (**a**) Representative live/dead staining images of HUVECs and NIH 3T3 cells after incubation with different hydrogel formulations, showing calcein-AM (green) and propidium iodide (PI, red) fluorescence. (**b**) CCK-8 assay of HUVEC viability after treatment with hydrogel extracts. Data are presented as mean ± SD (*n* = 3). (**c**) CCK-8 assay of NIH 3T3 viability after treatment with hydrogel extracts. Data are presented as mean ± SD (*n* = 3). (**d**) Representative photograph of the hemolysis test, with ddH_2_O and PBS used as positive and negative controls, respectively. (**e**) Quantitative analysis of the hemolysis ratio in different groups. Data are presented as mean ± SD (*n* = 3). (**f**) Representative H&E-stained sections of major organs, including the heart, liver, spleen, lung, and kidney, harvested from rats after subcutaneous implantation of different hydrogel formulations (*n* = 3). Data are presented as mean ± SD (*n* = 3); *** *p* < 0.001, ns not significant.

Live/dead staining showed strong calcein-AM fluorescence and minimal propidium iodide uptake in both HUVECs and NIH 3T3 cells cultured with all hydrogel formulations (Figure 4a), indicating high cell survival and no obvious acute cytotoxicity. Consistently, CCK-8 assays showed that cell viability remained above 90% in both cell types throughout the culture period (Figure 4b,c), indicating that the incorporation of CeO_2_NPs and VEGF did not compromise the cytocompatibility of the CSMA matrix at the tested concentrations.

Hemolysis testing produced marked erythrocyte disruption only in the distilled-water positive control, whereas supernatants from all hydrogel groups remained visually clear (Figure 4d). Quantitative analysis showed that the hemolysis ratios of all formulations were well below the generally accepted 5% threshold for blood-contacting biomaterials, indicating satisfactory hemocompatibility (Figure 4e).

Potential short-term systemic effects were further evaluated after subcutaneous implantation. H&E staining of the heart, liver, spleen, lung, and kidney revealed no detectable treatment-associated histopathological abnormalities relative to the control (Figure 4f). Together with the physiological and blood analyses described below, these findings indicate that no detectable adverse systemic effects were observed under the tested conditions during the 14-day observation period.

**Figure 5:**
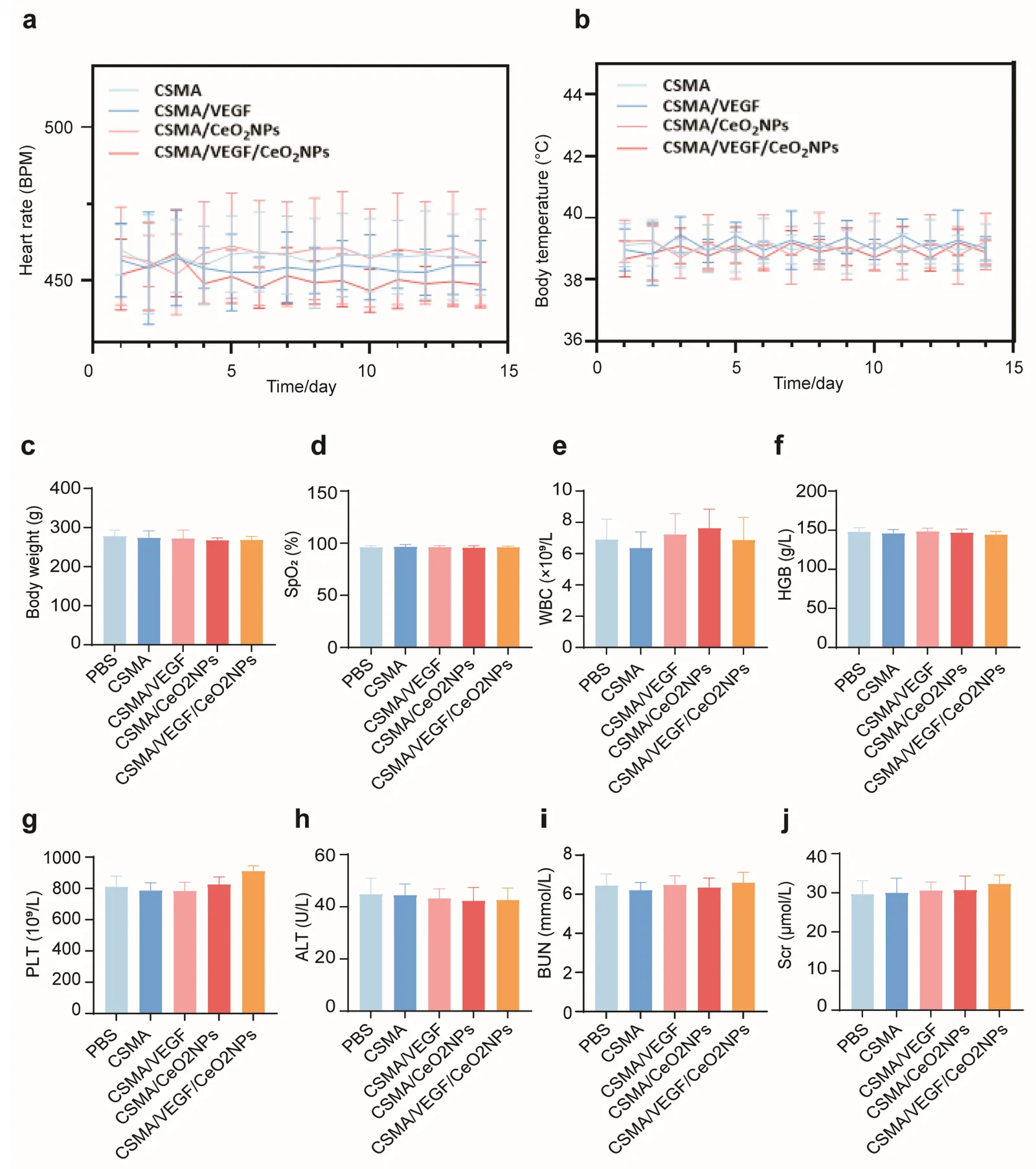
Physiological, hematological, and biochemical evaluation during the 14-day observation period following subcutaneous implantation. (**a**) Heart rate monitored daily over the 14-day observation period; no significant differences were observed among groups. (**b**) Body temperature monitored daily over the 14-day observation period; all groups remained within the normal physiological range. (**c**) Body weight measured at the experimental endpoint, showing comparable values across all groups. (**d**) Arterial oxygen saturation (SpO_2_) measured at the experimental endpoint, with no significant differences among groups. (**e**) White blood cell count (WBC) at the end of the 14-day observation period, with no significant between-group differences detected under the tested conditions. (**f**) Hemoglobin (HGB) levels at the end of the 14-day observation period, with no significant between-group differences detected under the tested conditions. (**g**) Platelet count (PLT) at endpoint, with all groups within the normal range. (**h**) Serum alanine aminotransferase (ALT) as a hepatic function marker, showing no significant elevation in any hydrogel group. (**i**) Blood urea nitrogen (BUN) as a renal function marker, comparable across all groups. (**j**) Serum creatinine (Scr) as a renal function marker, with no significant differences among groups. Groups: PBS, CSMA, CSMA/VEGF, CSMA/CeO_2_NPs, and CSMA/VEGF/CeO_2_NPs. Data are presented as mean ± SD (*n* = 6); no statistically significant differences were observed between groups (one-way ANOVA).

To further evaluate potential short-term systemic effects, physiological indicators and blood parameters were monitored following subcutaneous implantation (Figure 5). Heart rate and body temperature remained stable during the 14-day observation period, whereas body weight and SpO_2_ showed no significant between-group differences. White blood cell counts, hemoglobin levels, platelet counts, ALT, BUN, and serum creatinine were also comparable among groups at the assessed endpoints (Figure 5a–j). Collectively, no detectable adverse systemic effects were observed under the tested conditions during the 14-day observation period.

### 3.6. Antibacterial Activity Relevant to Infection-Associated Urinary Tract Applications

**Figure 6:**
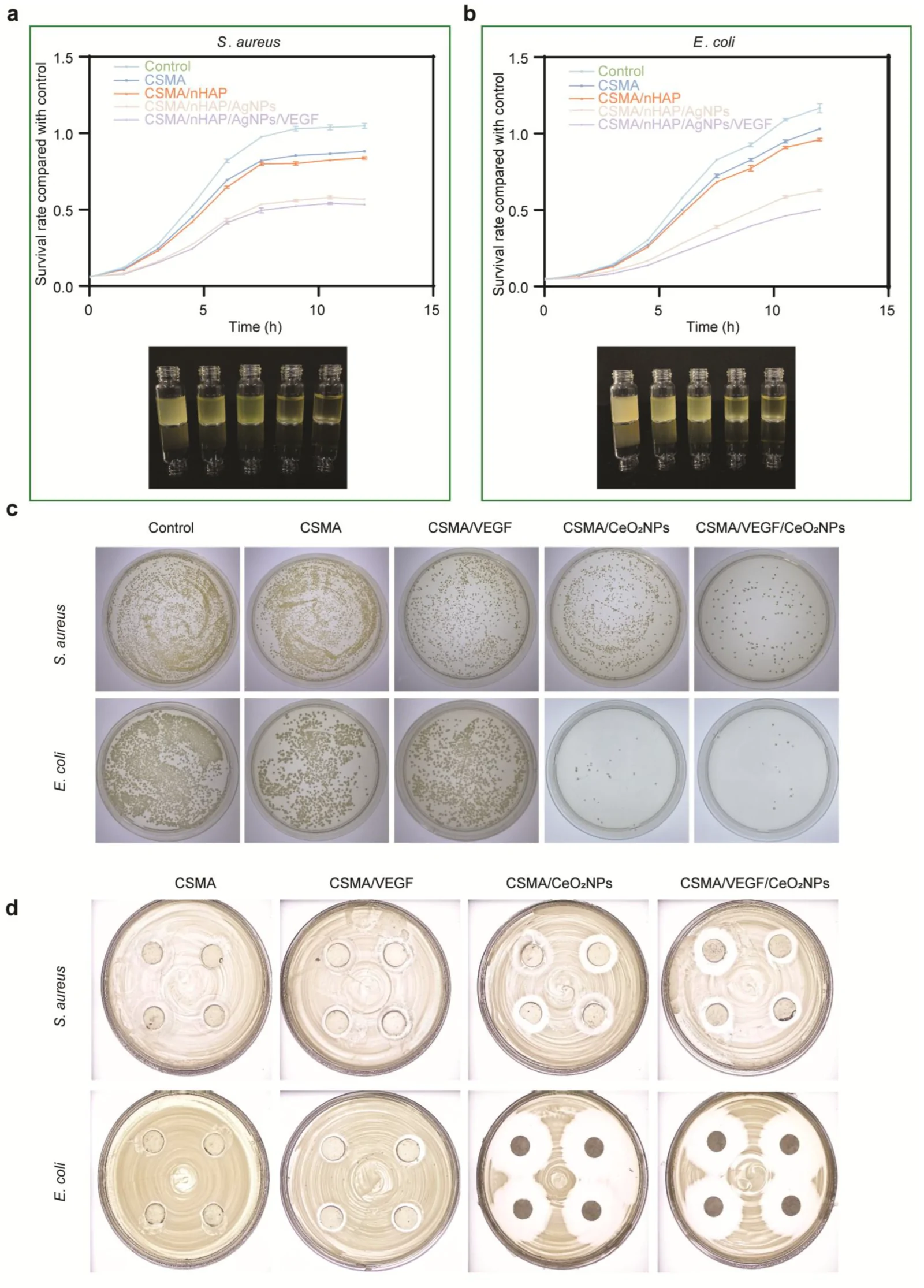
Antibacterial activity of CSMA-based hydrogels. (**a**,**b**) Time-course bacterial survival curves and corresponding culture photographs showing inhibition of *S. aureus* (**a**) and *E. coli* (**b**) by CSMA, CSMA/VEGF, CSMA/CeO_2_NPs, and CSMA/VEGF/CeO_2_NPs hydrogels, with an untreated bacterial suspension as the control. (**c**) Representative CFU plates showing residual colonies of *S. aureus* (**top**) and *E. coli* (**bottom**) after 24 h exposure to each material. (**d**) Disk diffusion assay showing inhibition zones produced by CSMA, CSMA/VEGF, CSMA/CeO_2_NPs, and CSMA/VEGF/CeO_2_NPs against *S. aureus* (**top**) and *E. coli* (**bottom**), with an untreated control included as a negative reference.

Because bacterial contamination can aggravate tissue injury and compromise biomaterial-assisted healing [28,29], we next investigated the antibacterial activity of the hydrogels against *S. aureus* and *E. coli* in the context of infection-associated urinary tract applications. Agar diffusion and bacterial survival assays revealed substantial differences among formulations (Figure 6a,b,d).

The control and pristine CSMA groups permitted rapid bacterial proliferation, with dense colony formation and only negligible inhibition zones, consistent with the weak intrinsic antibacterial activity of unmodified chitosan under these conditions. In contrast, the CeO_2_NPs-containing hydrogels markedly suppressed bacterial growth.

To quantify this effect, CFU assays were performed (Figure 6c). Relative to CSMA, both CeO_2_NPs-containing groups produced a pronounced reduction in viable counts of *E. coli* and *S. aureus*. These findings suggest that incorporation of CeO_2_NPs was primarily associated with the observed antibacterial activity. Notably, the CSMA/VEGF/CeO_2_NPs hydrogel showed antibacterial performance comparable to that of CSMA/CeO_2_NPs, indicating that VEGF incorporation did not significantly alter the antibacterial activity associated with CeO_2_NP incorporation.

Taken together, the agar diffusion, bacterial survival, and CFU results demonstrate that CeO_2_NPs incorporation substantially reduces bacterial burden in vitro. This antibacterial capacity is relevant to infection-associated urinary tract applications, where bacterial contamination may aggravate inflammatory injury and delay tissue recovery [30].

### 3.7. In Vivo Proof-of-Concept Evaluation Using Distinct Renal-Surface and Intravesical Models

To evaluate the in vivo performance of the hydrogels, both an infection-related injury setting and a renal ischemia–reperfusion injury (IRI) model were examined.

The two models involved distinct administration routes. The IRI model used direct renal-surface application during a surgically controlled procedure, whereas the ascending UTI model used catheter-based intravesical administration. These routes represent separate proof-of-concept applications and should not be interpreted as interchangeable.

The infection-related setting assessed whether the multifunctional hydrogel could alleviate tissue damage and inflammatory responses under bacteria-associated conditions, whereas the renal IRI model provided a biologically relevant platform for evaluating tissue protection and recovery in an injury-stressed renal microenvironment. Because renal IRI reproduces several pathological processes central to tissue repair, including acute parenchymal injury, inflammatory-cell infiltration, and remodeling-associated tissue disruption, it is particularly informative for assessing the in vivo protective effects of the hydrogel platform [31].

**Figure 7:**
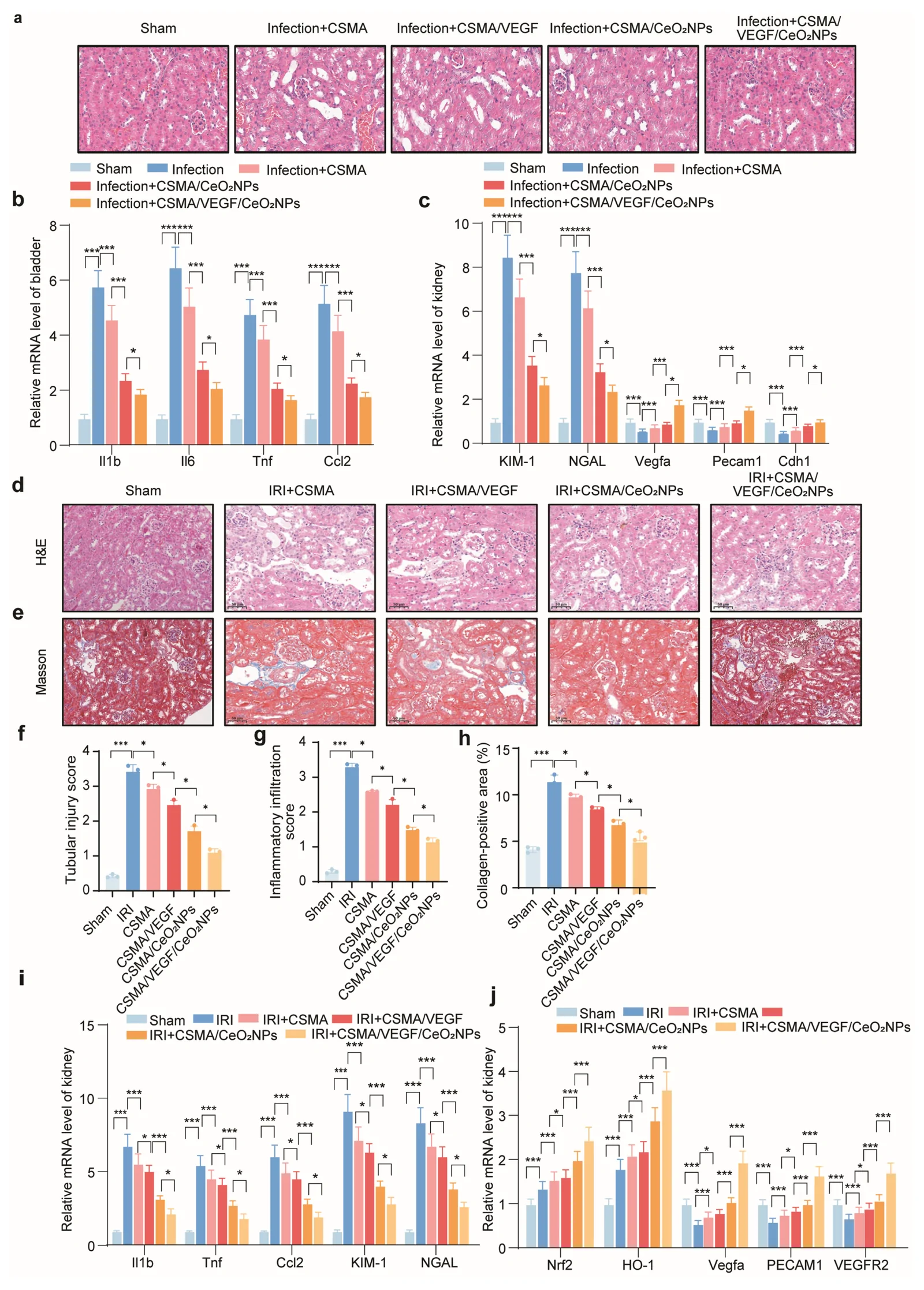
In vivo evaluation of hydrogels in infection-related repair and renal ischemia–reperfusion injury settings. (**a**) Representative histological observations of kidney tissues in the infection-related setting (*n* = 5 per group). (**b**) Relative mRNA expression of inflammation-related genes in bladder tissues from the infection-related setting. (**c**) Relative mRNA expression of renal injury-/repair-related genes in kidney tissues from the infection-related setting. (**d**) Representative H&E staining of kidney sections in the renal IRI model (*n* = 5 per group). (**e**) Representative Masson’s trichrome staining of kidney sections in the renal IRI model (*n* = 5 per group). (**f**) Quantitative analysis of tubular injury score. (**g**) Quantitative analysis of inflammatory infiltration score. (**h**) Quantitative analysis of collagen-positive area. (**i**) Relative mRNA expression of inflammatory and injury-related genes in kidney tissues from the renal IRI model. (**j**) Relative mRNA expression of vascularization- and repair-related genes in kidney tissues from the renal IRI model. Data are presented as mean ± SD,* *p* < 0.05, *** *p* < 0.001, ns not significant.

In the infection-related setting, representative histological observations of bladder and kidney tissues showed that infection induced apparent tissue damage and structural disturbance, whereas hydrogel treatment progressively alleviated these pathological changes in a formulation-dependent manner, with the CSMA/VEGF/CeO_2_NPs group showing the greatest improvement (Figure 7a). Among the treated groups, the CSMA/CeO_2_NPs and CSMA/VEGF/CeO_2_NPs formulations showed more favorable tissue morphology than CSMA alone. Consistently, qPCR analysis of infection-associated bladder inflammatory markers demonstrated that the expression levels of pro-inflammatory genes were markedly elevated after infection but were reduced after hydrogel treatment, with the CeO_2_NPs-containing groups showing the strongest suppressive effects (Figure 7b). In parallel, kidney mRNA analysis in the infection-related setting showed that renal injury-associated markers were elevated in the infection group and were correspondingly downregulated after treatment, whereas vascular- and repair-related genes were upregulated in the CSMA/VEGF/CeO_2_NPs group relative to untreated infection controls (Figure 7c). Together, these findings indicate that the composite hydrogel, particularly the CeO_2_NPs- and VEGF-containing formulation, can mitigate infection-associated tissue injury and help establish a more favorable local tissue microenvironment for recovery.

Histological examination in the renal IRI model further revealed marked differences in renal tissue injury among the groups. H&E staining showed that the injured kidneys displayed obvious tubular damage, structural disruption, and inflammatory-cell infiltration, whereas hydrogel treatment progressively improved tissue morphology (Figure 7d). Among the treatment groups, CSMA alone provided limited protection, while the addition of CeO_2_NPs and VEGF further alleviated renal injury. The CSMA/VEGF/CeO_2_NPs group showed the most favorable histological appearance, with better preservation of renal architecture and less apparent tissue damage than the other treated groups. Masson’s trichrome staining showed increased collagen deposition in the IRI group, whereas hydrogel-treated groups exhibited reduced collagen-positive staining, again with the CSMA/VEGF/CeO_2_NPs group showing the greatest reduction in collagen-positive area (Figure 7e).

Semi-quantitative analysis further corroborated these histological findings. Both tubular injury scores and inflammatory infiltration scores were markedly elevated following IRI but declined with hydrogel treatment, with the greatest improvement observed in the CSMA/VEGF/CeO_2_NPs group (tubular injury score: 3.415 ± 0.208 in IRI vs. 1.091 ± 0.114 in CSMA/VEGF/CeO_2_NPs, *p* < 0.05; inflammatory infiltration score: 3.289 ± 0.115 vs. 1.131 ± 0.127, *p* < 0.05; Figure 7f,g). Consistent with this pattern, quantification of collagen-positive area revealed a significant reduction from 11.693 ± 1.297% in the IRI group to 5.530 ± 0.463% in the CSMA/VEGF/CeO_2_NPs group (*p* < 0.05), suggesting that the multifunctional hydrogel effectively attenuated fibrosis-associated tissue remodeling in the injured kidney (Figure 7h). Renal RT-qPCR analysis further demonstrated that IRI substantially upregulated inflammatory and injury-related gene expression, which was suppressed by hydrogel treatment, most notably in formulations incorporating CeO_2_NPs and VEGF (Figure 7i). Conversely, the antioxidant-response genes Nrf2 and Ho-1, as well as the pro-angiogenic genes Vegfa, Pecam1, and Vegfr2, were upregulated to a greater extent in hydrogel-treated groups relative to the untreated IRI group, with the CSMA/VEGF/CeO_2_NPs formulation exhibiting the most favorable overall transcriptional profile (Figure 7j).

Overall, these in vivo findings indicate that the multifunctional hydrogel can alleviate infection-associated tissue injury, reduce inflammatory activation, and improve tissue repair-associated responses. In the renal IRI model, the hydrogel further attenuated renal tissue damage, inflammatory-cell infiltration, and collagen deposition, while favorably regulating injury-, inflammation-, and repair-related gene expression. Notably, these phenotype-level and targeted molecular findings suggested that CeO_2_NPs-containing hydrogel treatment may induce broader reprogramming of the local tissue microenvironment beyond the limited set of markers examined by histology and RT-qPCR. To further elucidate the molecular basis underlying these protective and pro-reparative effects, we next performed bulk RNA sequencing in both the infection and renal IRI models. This transcriptomic analysis was intended to determine whether the histologically observed tissue protection was accompanied by coordinated suppression of inflammatory programs and activation of repair-associated pathways at the global gene-expression level.

### 3.8. Transcriptomic Analysis and Experimental Validation Reveal Coordinated Regulation of Antigen Presentation, Metabolic Reprogramming, and Renal Structural Repair Following Multifunctional Hydrogel Treatment

**Figure 8:**
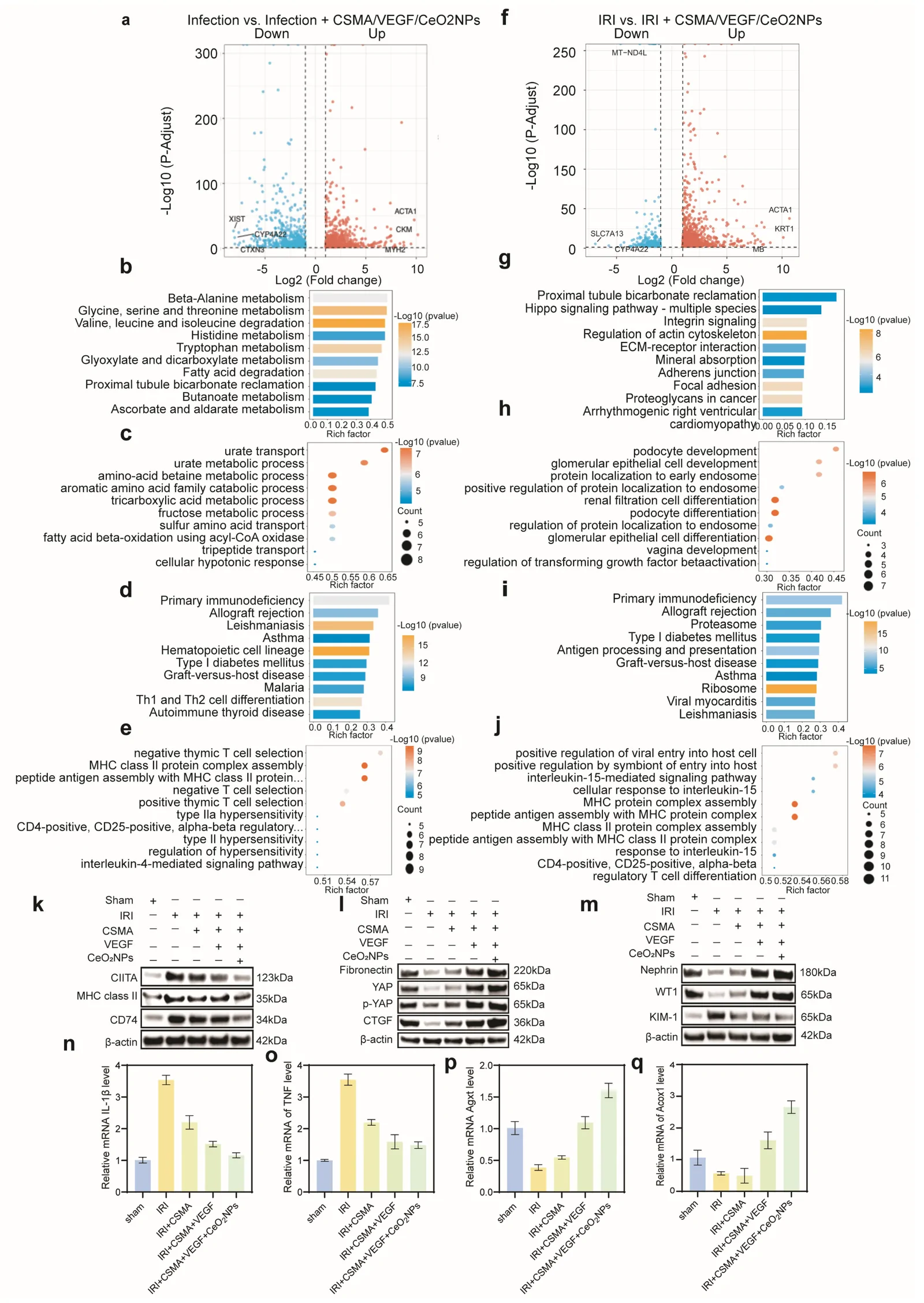
Transcriptomic analysis and experimental validation of metabolic reprogramming, MHC class II-associated antigen presentation, Hippo/YAP signaling, and renal structural markers in the infection and renal ischemia–reperfusion injury models following CSMA/VEGF/CeO_2_NPs treatment. (**a**) Volcano plot of DEGs in the infection model (untreated infection vs. CSMA/VEGF/CeO_2_NPs-treated). The dashed lines indicate the cutoff values for fold change and statistical significance. (**b**) KEGG pathway enrichment analysis of upregulated DEGs (infection model). (**c**) Gene Ontology (GO) biological process enrichment analysis of upregulated DEGs. (**d**) KEGG pathway enrichment analysis of downregulated DEGs. (**e**) GO biological process enrichment analysis of downregulated DEGs. (**f**) Volcano plot of DEGs in the renal IRI model (untreated IRI vs. CSMA/VEGF/CeO_2_NPs-treated). (**g**) KEGG pathway enrichment analysis of upregulated DEGs (IRI model). (**h**) GO biological process enrichment analysis of upregulated DEGs. (**i**) KEGG pathway enrichment analysis of downregulated DEGs. (**j**) GO biological process enrichment analysis of downregulated DEGs. (**k**) Representative Western blot images showing the expression of MHC class II antigen presentation-related proteins, including CIITA, MHC class II, and CD74, in renal tissues from the Sham, IRI, IRI + CSMA, IRI + CSMA + VEGF, and IRI + CSMA + VEGF + CeO_2_NPs groups. (**l**) Representative Western blot images showing the expression of fibrosis- and Hippo/YAP pathway-related proteins, including Fibronectin, YAP, p-YAP, and CTGF. (**m**) Representative Western blot images showing the expression of renal structural and tubular injury markers, including Nephrin, WT1, and KIM-1. (**n**) Relative mRNA expression of IL-1β in renal tissues determined by qPCR. (**o**) Relative mRNA expression of TNF in renal tissues determined by qPCR. (**p**) Relative mRNA expression of Agxt determined by qPCR. (**q**) Relative mRNA expression of Acox1 determined by qPCR. Data are presented as mean ± SD (*n* = 3). Statistical significance was determined by one-way ANOVA followed by Tukey’s multiple-comparison test.

To elucidate the molecular mechanisms underlying the renoprotective effects of the multifunctional hydrogel, bulk RNA sequencing was performed on renal tissues from both the infection model and the ischemia–reperfusion injury (IRI) model, in each case comparing untreated injury with the CSMA/VEGF/CeO_2_NPs-treated group. Differential expression analysis revealed substantial transcriptomic remodeling after treatment in both models (infection model, Figure 8a–e; IRI model, Figure 8f–j), indicating that the histological protection was accompanied by broad changes in gene expression.

In the infection model, functional enrichment analysis showed that upregulated genes were predominantly associated with metabolic pathways, including glycine, serine and threonine metabolism, fatty acid metabolism, beta-alanine metabolism, and urate metabolism (Figure 8b,c). Among these differentially expressed genes were key enzymes of amino acid, lipid, and purine metabolism, including AGXT, ACOX1, and XDH. These findings indicate that treatment promotes restoration of renal metabolic programs following infection-associated injury.

Conversely, downregulated genes in the infection model were enriched in immune-related pathways, including antigen processing and presentation, MHC class II-associated processes, hematopoietic cell lineage, and the allograft-rejection gene set (which shares many antigen-presentation and MHC class II genes rather than indicating transplant rejection per se) (Figure 8d,e). The same immune-suppressive pattern was observed in the IRI model (Figure 8i,j), indicating attenuation of maladaptive antigen presentation and immune activation after treatment in both injury settings.

In the IRI model, enrichment was also observed for pathways associated with tissue repair and structural remodeling, including Hippo signaling, ECM-receptor interaction, focal adhesion, podocyte development, glomerular epithelial cell differentiation, and renal filtration processes (Figure 8g,h). Because the IRI model is predominantly a tubular injury, these glomerular-annotated terms are interpreted cautiously as part of a broader renal structural-repair signature rather than as evidence of a podocyte-specific mechanism; overall, they suggest that treatment supports recovery of renal structural integrity following ischemic injury.

To validate these findings, representative targets were examined at the protein and mRNA levels in independent renal tissue samples from the IRI model. Western blotting showed that IRI markedly increased the antigen-presentation proteins CIITA, MHC class II, and CD74, whereas CSMA/VEGF/CeO_2_NPs treatment markedly reduced them (Figure 8k), corroborating the transcriptomic suppression of MHC class II-mediated antigen presentation. Consistent with the enrichment of Hippo and ECM-remodeling pathways, treatment decreased CTGF and fibronectin and increased phospho-YAP relative to total YAP (Figure 8l). The renal structural markers nephrin and WT1 were restored and the tubular injury marker KIM-1 was reduced after treatment (Figure 8m), consistent with improved preservation of renal structural integrity. At the mRNA level, qPCR showed that the pro-inflammatory genes Il1b and Tnf, which were elevated by IRI, were markedly downregulated after treatment (Figure 8n,o), in agreement with the suppression of immune-related pathways. Finally, qPCR confirmed significant upregulation of the metabolic enzymes AGXT and ACOX1 in treated kidneys (Figure 8p,q), consistent with the transcriptomic evidence of metabolic restoration.

Together, these results indicate that CSMA/VEGF/CeO_2_NPs treatment is associated with a coordinated molecular program comprising attenuated antigen presentation, restored metabolic homeostasis, activation of repair-associated signaling, and preservation of renal structural markers. These molecular changes are compatible with the literature-informed hypothesis that CeO_2_NPs may exert redox-modulating effects. However, the transcriptomic and validation data provide only indirect support and do not directly demonstrate ROS scavenging or Ce^3+^/oxygen-vacancy-mediated redox activity.

## 4. Discussion

The present study demonstrates that a multifunctional CSMA-based hydrogel can simultaneously provide antibacterial protection, pro-angiogenic stimulation, and tissue-protective activity within a single injectable platform. Importantly, these biological functions were achieved without compromising the physicochemical properties of the hydrogel, including swelling behavior, mechanical stability, and viscoelastic recovery.

CeO_2_NPs and VEGF appeared to exert complementary therapeutic effects. CeO_2_NPs-containing hydrogels exhibited sustained antibacterial activity and were associated with broad regulation of injury-associated molecular pathways, whereas VEGF promoted angiogenesis and tissue regeneration. Previous studies suggest that mixed-valence CeO_2_ nanoparticles may exhibit redox-modulating activity. In the present study, treatment with the complete formulation was associated with increased expression of the antioxidant-response genes Nrf2 and HO-1 (Figure 7j); however, this association provides only indirect support and does not directly demonstrate ROS scavenging or Ce^3+^/oxygen-vacancy-mediated redox activity. The combination of these functionalities resulted in superior therapeutic performance compared with single-component formulations, as evidenced by improved histological outcomes, reduced inflammatory responses, and enhanced expression of repair-associated genes in vivo.

From a biological perspective, the results suggest that the multifunctional hydrogel can establish a local microenvironment favorable for tissue recovery. In vitro, the hydrogel supported HK-2 tubular epithelial cell migration, NIH 3T3 fibroblast migration and matrix-remodeling capacity, HUVEC tube formation, and angiogenesis-related responses, indicating that the material can promote several cellular events relevant to tissue protection and remodeling. At the same time, CeO_2_NPs-containing formulations effectively reduced bacterial burden in vitro, suggesting an additional protective advantage for use in contamination-prone repair settings. These combined features are particularly meaningful for renal biomaterial development because renal tissue injury is often accompanied by inflammation, oxidative stress, microvascular dysfunction, and impaired local healing conditions [32,33,34,35].

In vivo, the renal ischemia–reperfusion injury model provided further support for the tissue-protective performance of the hydrogel in a urologically relevant organ context. Hydrogel treatment, particularly with the CSMA/VEGF/CeO_2_NPs formulation, was associated with improved histological appearance, reduced inflammatory infiltration, and attenuated tissue injury. These findings, together with the in vitro antibacterial and pro-angiogenic results, indicate that the multifunctional hydrogel can beneficially modulate the local injury microenvironment and support renal tissue recovery through the coordinated action of structural, antimicrobial, and regenerative cues [36]. Transcriptomic profiling of injured renal tissue further indicated that treatment suppressed antigen-presentation and immune-activation programs while restoring metabolic and tissue-repair pathways. These transcriptomic shifts were corroborated at both the gene and protein levels by RT-qPCR and Western blotting on independent renal tissue samples, strengthening the molecular evidence for the proposed tissue-protective and pro-reparative mechanism.

Overall, the present findings support a biomaterial design strategy in which structural reinforcement, antibacterial protection, and pro-regenerative stimulation are integrated within a single injectable hydrogel system [37]. By combining rapid in situ gelation, sustained antibacterial activity, pro-angiogenic bioactivity, and coordinated regulation of immune, metabolic, and tissue-repair pathways, the CSMA/VEGF/CeO_2_NPs hydrogel provides a multifunctional framework for renal tissue repair and protection. These findings support further investigation of multifunctional biomaterials for ischemic renal injury and other inflammation-associated renal disorders. No detectable cytotoxicity or hemolysis was observed in the assays performed, and no detectable adverse systemic effects were observed under the tested conditions during the 14-day observation period.

## 5. Limitations of the Study

First, all experiments used a single chitosan lot and CSMA batch, so cross-batch reproducibility was not established; the dissolved versus colloidal fraction of the precursor was also not directly quantified. Batch qualification (DS by NMR, gelation time, compressive modulus, swelling ratio) will be needed before translational development.

Second, enzymatic degradation was not assessed. Instead, the mass-loss assay primarily reflects the elution of soluble fragments rather than lysozyme-mediated chain scission, which is the predominant degradation pathway in vivo. Therefore, future studies should include lysozyme-containing degradation assays to better evaluate the enzymatic degradation behavior of the hydrogel. In addition, differences in the molecular weight and degree of deacetylation of chitosan may influence the number and accessibility of amino groups available for methacrylation, thereby affecting methacrylation efficiency, crosslinking density, gelation kinetics, mechanical strength, swelling behavior, and degradation rate. These factors should also be systematically investigated in future studies.

Third, the proposed Ce^4+^-mediated antibacterial effect and Ce^3+^/oxygen-vacancy-associated redox activity are literature-informed hypotheses supported by indirect compositional and functional evidence. Neither mechanism was directly demonstrated in this study. Bacterial ROS and membrane-integrity assays, direct ROS-modulation assays, and independent characterization of oxygen vacancies would be required to test these hypotheses.

Fourth, transcutaneous crosslinking of the bladder-instilled hydrogel in the infection model lacks direct confirmation that adequate photon flux reached the target tissue; ex vivo light-transmission or terminal-retrieval studies are needed to validate this route.

Fifth, the 14-day window captures only acute effects; long-term cerium biodistribution and accumulation (CeO_2_NPs are expected to be more persistent than VEGF) were not assessed, and the photoinitiator LAP was not tested in isolation. Extended-timepoint biodistribution studies are needed before clinical translation. The clearance, persistence, and long-term biological fate of CeO_2_ nanoparticles remain uncertain. There is the possibility of local retention and accumulation in reticuloendothelial organs such as the liver and spleen, as well as potential risks associated with chronic cerium retention. Quantitative biodistribution and clearance studies at extended time points are required in future work.

Sixth, this is a proof-of-concept model with a narrow translational scope: direct renal-surface application is applicable to surgically controlled procedures, such as partial nephrectomy or transplant back-table preparation. By contrast, catheter-based intravesical administration represents a distinct delivery strategy intended for lower urinary tract applications and should not be considered interchangeable with renal-surface administration.

## 6. Conclusions

In summary, we developed a multifunctional injectable CSMA-based hydrogel incorporating VEGF and nanosized CeO_2_ nanoparticles for renal tissue protection and repair. The hydrogel exhibited favorable physicochemical properties, sustained release behavior, favorable in vitro cytocompatibility and hemocompatibility under the tested assay conditions, potent antibacterial activity, and robust pro-angiogenic capability.

In vivo studies demonstrated that the CSMA/VEGF/CeO_2_NPs hydrogel effectively alleviated renal ischemia–reperfusion injury, reduced inflammatory infiltration and tissue damage, and improved renal histological outcomes. Transcriptomic analysis combined with qPCR and Western blot validation further revealed that treatment suppressed MHC class II-associated antigen presentation pathways, restored metabolic programs involving AGXT and ACOX1, regulated Hippo/YAP- and ECM-related signaling, and preserved renal structural markers including nephrin and WT1.

These findings suggest that treatment-associated renal recovery was accompanied by coordinated changes in immune responses, metabolic homeostasis, and tissue repair pathways. Furthermore, no detectable adverse systemic effects were observed under the tested experimental conditions during the 14-day observation period. This study provides insight into potential mechanisms associated with the renal protective effects of mixed-valence CeO_2_ nanoparticles. Based on the previous literature, together with the compositional characterization and functional observations obtained here, the Ce^4+^-predominant surface composition may contribute to antibacterial activity, while the detected Ce^3+^ surface population and associated oxygen vacancies may participate in redox modulation in the host tissue microenvironment.

## Data Availability

The data presented in this study are available on request from the corresponding author.
